# A Robust Silicone Rubber Strip-Based Triboelectric Nanogenerator for Vibration Energy Harvesting and Multi-Functional Self-Powered Sensing

**DOI:** 10.3390/nano12081248

**Published:** 2022-04-07

**Authors:** Taili Du, Bin Ge, Anaeli Elibariki Mtui, Cong Zhao, Fangyang Dong, Yongjiu Zou, Hao Wang, Peiting Sun, Minyi Xu

**Affiliations:** 1 Dalian Key Lab of Marine Micro/Nano Energy and Self-Powered Systems, Marine Engineering College, Dalian Maritime University, Dalian 116026, China; dutaili@dlmu.edu.cn (T.D.); gebin@dlmu.edu.cn (B.G.); mtyellie93@gmail.com (A.E.M.); zhaocong@dlmu.edu.cn (C.Z.); dongfangyang@dlmu.edu.cn (F.D.); zouyj0421@dlmu.edu.cn (Y.Z.); hao8901@dlmu.edu.cn (H.W.); 2 Collaborative Innovation Research Institute of Autonomous Ship, Dalian Maritime University, Dalian 116026, China

**Keywords:** silicone rubber strip, triboelectric nanogenerator, vibration sensor, energy harvesting

## Abstract

Vibration is a common phenomenon in various fields which can not only indicate the working condition of the installation, but also serve as an energy source if it is efficiently harvested. In this work, a robust silicone rubber strip-based triboelectric nanogenerator (SRS-TENG) for vibration energy harvesting and multi-functional self-powered sensing is proposed and systematically investigated. The SRS-TENG consists of a silicone rubber strip and two aluminum electrode layers supported by polylactic acid (PLA), and acts as a sustainable power source and vibration frequency, amplitude and acceleration sensor as well. The soft contact between the aluminum electrode and silicone rubber strip makes it robust and stable even after 14 days. It can be applied in ranges of vibration frequencies from 5 to 90 Hz, and amplitudes from 0.5 to 9 mm, which shows it has advantages in broadband vibration. Additionally, it can achieve lower startup limits due to its soft structure and being able to work in multi-mode. The output power density of the SRS-TENG can reach 94.95 W/m^3^, matching a resistance of 250 MΩ, and it can light up more than 100 LEDs and power a commercial temperature sensor after charging capacitors. In addition, the vibration amplitude can be successfully detected and displayed on a human–machine interface. Moreover, the frequency beyond a specific limit can be distinguished by the SRS-TENG as well. Therefore, the SRS-TENG can be utilized as an in situ power source for distributed sensor nodes and a multifunctional self-powered vibration sensor in many scenarios.

## 1. Introduction

With the development in autonomous/smart technologies and distributed, interconnected and self-powering sensor networks for Internet of Things (IoT) applications [1,2], a huge amount of distribution sensors are essential for the intellectualization of many different fields [3]. Meanwhile, vibration is a very common form of mechanical energy in many machines, vehicles, and structures which carries relevant information about their performance but which is otherwise irreversibly wasted without any further use [4]. Most distributed sensors should be powered through cables or by batteries. However, vast sensor nodes lead to complex arrangements and high cost in system design and construction because of large amounts of power and signal cables [5]. Moreover, battery-powered approaches also face serious challenges due to short battery life cycles and the pollution problems caused by battery aftertreatment [6,7]. Therefore, if vibration energy can be effectively collected and converted into electrical power, it will be beneficial for the in situ energy supply of massive distributed sensors [8]. It would be of great benefit if the vibration sensing information could also be obtained as well as simple energy harvesting. In brief, vibration energy harvesting and self-powered vibration sensing are prospective choices in the era of IoT in many application scenarios [9].

Furthermore, with triboelectric nanogenerators (TENGs), it is possible to make this vision a reality. TENGs, invented by Professor Zhong Lin Wang in 2012, work based on a combination of electrification effects and electrostatic induction between triboelectric materials [10,11,12]. TENGs are considered promising for wide applications such as self-powered sensors [13], nano/micropower [10], high-voltage energy [14], and energy-harvesting systems [5,15,16]. Recently, vibration energy harvesting and sensing based on TENGs have attracted great interest due to the advantages of high efficiency, low cost, compact size [17], etc. Generally, the reported vibration energy harvesters based on TENGs mainly include spring-assisted or similar resonant-type vibration harvesters, and non-resonant ones. The harmonic-type TENG can produce maximum electrical output within resonant frequencies [18,19]. However, the application of the harmonic-type TENG is limited in wide vibration ranges because the energy performance significantly decreases when the vibration is out of its sharp resonant frequency. Therefore, some multifrequency resonance-type TENGs [20] and non-resonant bouncing-ball-type TENGs [21] are reported for enhancing energy-harvesting performance in wide vibration ranges. However, there are still problems demanding prompt solutions, for example, high output performance is achieved only around several resonant frequencies, and the bouncing-ball-type model can only work under contact-separation mode. Therefore, the design of a TENG with broad frequency response and multi-mode capability should be highly considered.

In this work, a robust silicone rubber strip-based triboelectric nanogenerator (SRS-TENG) for broadband vibration energy harvesting and multi-functional vibration sensing is proposed. The SRS-TENG in a rectangular prism is composed of two conductive aluminum electrodes supported by PLA and a non-conductive silicone rubber strip with its ends fixed at the half-height of the rectangular prism. The SRS-TENG was tested under two different conditions. Firstly, it was tested under varying amplitudes with fixed vibration frequency. The SRS-TENG effectively detected vibration amplitudes from 0.5 to 9 mm under the vibration frequency of 20 Hz. Secondly, it was tested under varying frequencies with fixed vibration amplitudes. With a vibration amplitude of 1 mm, the SRS-TENG was capable of detecting a vibration frequency from 5 to 90 Hz. Moreover, it was able to light up more than 100 LEDs and power a temperature sensor successfully after charging a capacitor when working at a vibration frequency of 30 Hz. Therefore, the SRS-TENG has potential applications in self-powered vibration sensing and vibration energy harvesting with broadband vibrations.

## 2. Results and Discussion

### 2.1. Structure and Working Mechanism of the SRS-TENG

**Figure 1:**
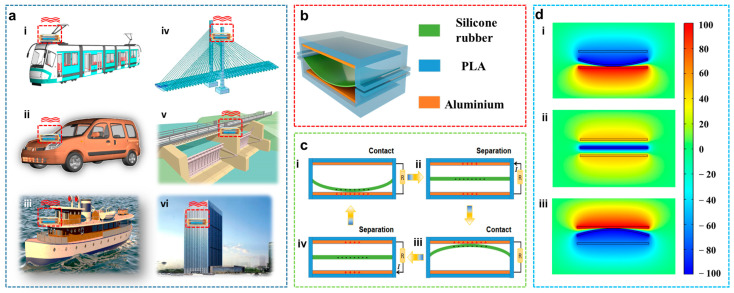
Application scenario, structure and working principle of SRS-TENG. (**a**) SRS-TENG application in various scenarios, such as (**i**) metro, (**ii**) vehicle, (**iii**) ship, (**iv**) bridge, (**v**) hydroelectric power plant, and (**vi**) building. (**b**) The structure and (**c**) (**i**–**iv**) working mechanism of the SRS-TENG; (**d**) (**i**–**iii**) the potential distribution of two parallel electrodes at different states calculated by COMSOL Multiphysics (COMSOL Inc., Stockholm, Sweden).

As shown in Figure 1a, the SRS-TENG can be used as a universal vibration energy harvesting and vibration sensing device in a variety of applications including metro, vehicle, ship, bridge, hydroelectric power plant, building, etc. The schematic diagram of the SRS-TENG is illustrated in Figure 1b. It consists of two conductive aluminum electrodes supported by the PLA and a silicone rubber strip in the rectangular prism. The two ends of the silicone rubber strip are fixed at the mid-height of the rectangular prism so that the silicone rubber strip is hung between the two aluminum electrodes. The air gap between the aluminum electrodes and silicone rubber strip is to facilitate the contact and separation between them.

The detailed working mechanism of SRS-TENG is demonstrated in Figure 1c. In the initial state, which is shown in Figure 1(ci), the silicone rubber strip makes contacts with the bottom electrode due to its elastic deformation caused by the external vibration excitation. Due to their different electronegativities, the mechanical contact between the silicone rubber strip and the aluminum electrodes leads to electron-enhanced and electron-depleted regions on adjacent surfaces and therefore ensuing electric fields. Then, with the upward oscillation of the silicone rubber strip, the electrons move from the top electrode to the bottom one because of the potential difference between them, which is illustrated in Figure 1(cii). When the silicone rubber strip makes contact with the top layer, a new equilibrium is achieved, as shown in Figure 1(ciii). Then, the silicone rubber begins to move downwards, which makes the electrons move from the bottom electrode to the top one. Thus, a current in Figure 1(civ) contrary to that in Figure 1(cii) is generated. As a result, the vibration energy is effectively converted to electrical energy.

Furthermore, the simulation result of the electrostatic field distribution of the SRS-TENG by COMSOL Multiphysics is depicted in Figure 1d, which matches well with the working mechanism. Besides the contact-separation (C-S) mode, the SRS-TENG can work in non-contact (N-C) mode as well. The working mechanism of the N-C mode is shown in Figure S1 in the Supplementary Materials. The output performance of both modes was examined, and the results are discussed in the following section.

### 2.2. Theoretical Analysis

Based on the theory of the contact-mode freestanding TENG [11,22,23,24], the equation for SRS-TENG is elaborated as:

(1) V=−1CQ+VOC=−d0+hagε0SQ+2σ(hag2−ω)ε0,

where *V_OC_* is the open-circuit voltage, *Q* is the transferred charge, *C* is the capacitance, *d*_0_ is the thickness of the silicone rubber strip, *h_ag_* is the height of the air gap between the electrode and the strip, *ε*_0_ is the dielectric constant in vacuum, *S* is the size of the electrode, *ω* is the separation distance between the electrode and the strip along the y-coordinate due to elastic deformation, and *σ* is the charge density.

**Figure 2:**
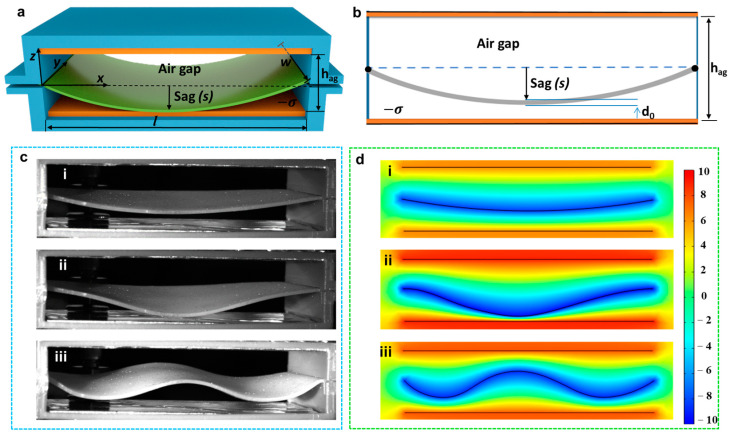
Analysis of the movement of silicone rubber strip. (**a**) The coordinate system of the SRS-TENG shown in the 3D structure; (**b**) parameters of the SRS-TENG; (**c**) silicone rubber strip taken by high-speed camera and (**d**) its corresponding simulation result by COMSOL Multiphysics at vibration frequencies of (**i**) 10 Hz, (**ii**) 30 Hz and (**iii**) 50 Hz, respectively.

As depicted in Figure 1c and Figure S1, the SRS-TENG can work in both C-S mode and N-C mode. In order to represent the working condition of the SRS-TENG, the deformation status of the silicone rubber strip is defined, where the distance of separation from the origin of the coordinate is expressed as sag (s), as shown in Figure 2a. The sag (s) of the silicone rubber strip can also be stated as the length from the deepest point of the full expansion strip to the origin of the coordinate. In addition, parameters such as the air gap *h_ag_* of the SRS-TENG unit, the width *w* and thickness *d*_0_ of the strip are demonstrated in the 3D structure and cross-section of the SRS-TENG displayed in Figure 2a,b.

The SRS-TENG works based on the alternative movement of the silicone rubber strip due to the triggering of the vibration source [25,26]. Therefore, the sag (*s*) or the length (*l*), width (*w*), thickness (*d*_0_) of the strip, and the height of the rectangular prism are the key parameters that influence the oscillation behavior of the strip, as well as the electrical output of the SRS-TENG.

On basis of the theoretical analysis, the movements of the silicone rubber strip under different external vibrations were investigated by applying a high-speed camera (FATCAM Mini UX50, Photron, Tokyo, Japan), as shown in Figure 2c, and simulated through COMSOL Multiphysics (COMSOL Inc., Stockholm, Sweden), as illustrated in Figure 2d. At a constant amplitude of 1 mm with different vibration frequencies, the strip behaves in different vibration forms as the vibration frequency increases. Figure 2(ci–ciii) show the strip wave status at vibration frequencies of 10, 30 and 50 Hz, respectively. The more detailed moving status of the strip caused by different external vibrations is shown in Video S1 in the Supplementary Materials. As shown in Figure 2(di–diii), the simulation results agree well with the movement of the strip in the SRS-TENG. It was observed that the strip wave amplitude decreases and the number of cycles increases with the increasing vibration frequency because the frequency of a wave is related to its wavelength expressed by the equation *f_w_* = *v_w_*/*λ_w_*, where *v_w_* and *λ_w_* represent the wave speed and wavelength, respectively [27].

### 2.3. Working Performance of SRS-TENG

A testing system, which is shown in Figure S2 in the Supplementary Materials, was built to test the working performance of the SRS-TENG. The SRS-TENG was mounted on an electrodynamic shaker (JZK-20, SINOCERA, Suzhou, China), which served as the external vibration source. The shaker, which is driven by an external amplifier (YE5872A, SINOCERA, Suzhou, China) that receives vibration signal from a function generator (YE1311, SINOCERA, Suzhou, China), can generate various forms of vibration with adjustable frequency and amplitude. The maximum acceleration of the device is monitored by a commercial accelerometer (KS96.100, MMF, Radebeul, Germany) and analyzed by a dynamic signal analyzer. The relationship is governed by the harmonic motion equation which can be derived from calculus theory [21,23],

(2) $$\begin{array}{c} y_{b}=A\sin(\omega t+\varphi )\\ a_{m}=A\omega^{2}=A{\left(2\pi f\right)}^{2} \end{array}$$

where *a_m_*, *f*, and *A* are the maximum vibration acceleration, vibration frequency and vibration amplitude, respectively.

**Figure 3:**
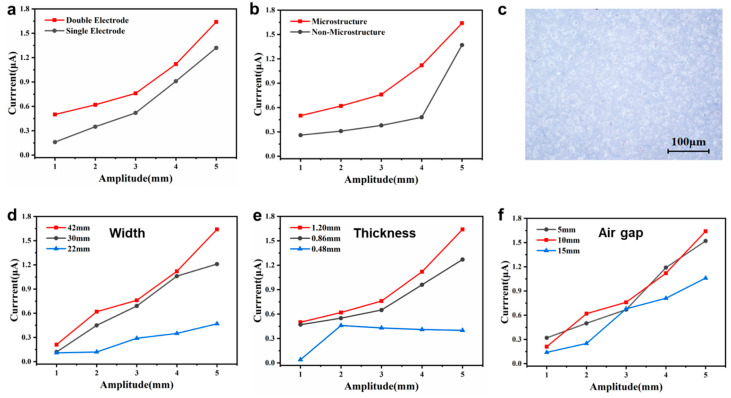
The impacts of different working modes and parameters on the output performance of the SRS-TENG. Current performance comparison between the SRS-TENGs (**a**) working at double electrode and single electrode modes, and (**b**) with or without surface treatment of the strip. (**c**) Surface microstructure of silicone rubber polished by sandpaper. (**d**) Current performance comparison among the SRS-TENGs with different widths of 22, 30, and 42 mm, (**e**) different thicknesses of 0.48, 0.86, and 1.2 mm, and (**f**) different air gaps of 5, 10 and 15 mm.

In order to study the impacts of different working modes and parameters on the output performance of the SRS-TENG, the SRS-TENGs with single and double electrode(s), with or without rubber strip modification, different rubber strip widths and thicknesses, and different air gaps, are systematically studied. As shown in Figure 3a, the output current of SRS-TENG with a double electrode mode had a 50% improvement and showed a more stable performance compared to that with a single electrode. The output voltage and transferred charge comparison between the single and double electrode(s) modes of the SRS-TENG are illustrated in Figure S3 in the Supplementary Materials. The output performance of the SRS-TENG with a silicone rubber strip surface modified by sandpaper indicated that the output current, voltage and transferred charge increased by 50%, 40% and 20%, respectively, compared to that with the original strip, as demonstrated in Figure 3b and Figure S4 in the Supplementary Materials. Referring to [28,29], the contact electrification of the TENG is realized by the friction or contact nanomaterials in the field of the electron scope. Additionally, the microstructure after the surface modification of the strip is shown in Figure 3c. Therefore, the performance improvement after surface modification was due to the increase in the nanostructure contact area between the rubber strip and the electrode, contributing to a higher output performance of the SRS-TENG [30,31,32].

Figure 3d and Figure S5 in the Supplementary Materials show that the output performance of the SRS-TENG increases as the width of the strip increases under the frequency of 20 Hz and varying amplitudes. This is also because the contact area between the nanostructures of the strip and electrode increases, thereby leading to high output performance. In addition, strips with various thicknesses are realized by adjusting the acceleration, speed and time of the spin-coater, as shown in Table S1 in the Supplementary Materials. Three strips with a thickness of 0.48, 0.86 and 1.2 mm were fabricated and investigated.

As depicted in Figure 3e and Figure S6 in the Supplementary Materials, the output performance increased in the wake of increasing the thickness of the strip. The reason for that is the thicker the strip, the heavier the strip, which results in a larger contact force between the strip and the electrode. Moreover, the SRS-TENGs with an air gap of 5, 10 and 15 mm were fabricated to study the effect of the *h_ag_* on the output performance of the SRS-TENG. Figure 3f and Figure S7 in the Supplementary Materials show the maximum output of the SRS-TENG with an air gap of 10 mm compared to the air gaps of 5 and 15 mm. The reason for this trend is that small air gap limits the separation distance between the strip and the electrode, and an excessive air gap causes ineffective contact. Hence, the performance of the SRS-TENG degrades in both cases.

Therefore, an SRS-TENG with strip surface modification, thickness of 1.2 mm, width of 42 mm, air gap of 10 mm, and double electrodes, was determined to carry out the output performance test.

According to the theoretical analysis, both vibration amplitude and frequency had a vital impact on the working performance of SRS-TENG. So, the output performance of the SRS-TENG under the condition of variable amplitudes of 0.5–9 mm and frequencies of 5–90 Hz were comprehensively carried out. However, it should be noted that the electrodynamic shaker will not work at excessive acceleration, which may result in the failure of the shaker according to the manufacturer’s recommendation; therefore, vibration at a large amplitude at high frequency could not be carried out.

**Figure 4:**
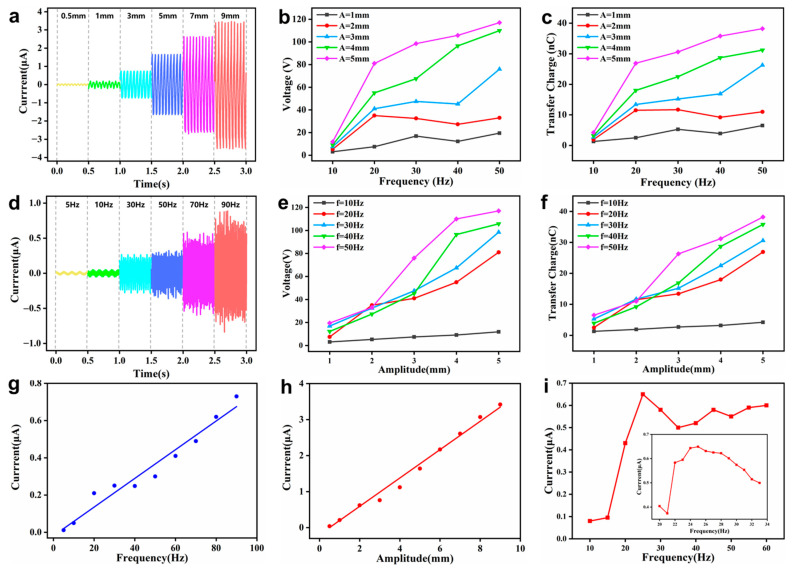
Electrical output performance of the SRS-TENG. (**a**) Short-circuit current with different vibration amplitudes under a vibration frequency of 20 Hz. (**b**) Open-circuit voltage and (**c**) transferred charge with different vibration amplitudes under different fixed frequencies. (**d**) Short-circuit current with different vibration frequencies under a vibration amplitude of 1 mm. (**e**) Open-circuit voltage and (**f**) transferred charge with different vibration frequencies under different fixed amplitudes. (**g**) Linear relationship between the short-circuit current and vibration frequency at a fixed vibration amplitude of 1 mm. (**h**) Linear relationship between the short-circuit current and different vibration amplitude at a fixed vibration frequency of 20 Hz. (**i**) Short-circuit current under a vibration acceleration of 20 m/s^2^.

As illustrated in Figure 4a, the short-circuit current increased from 0.1 to 3.1 µA as the vibration amplitude increased from 0.5 to 9 mm under the fixed vibration frequency of 20 Hz, which is a typical representative within the scope of various fixed frequencies from 5–90 Hz. However, as exhibited in Figure 4b,c, the relationship of open-circuit voltage and transferred charge varying with amplitude at different fixed frequencies did not show a positive linear correlation.

Moreover, the output performance under different vibration frequencies, which changed from 5 to 90 Hz, was also explored. As depicted in Figure 4d, the short-circuit current increased from 0.1 to 0.8 μA with the vibration frequency from 5 to 90 Hz and amplitude fixed at 1 mm. The variation tendency of open-circuit voltage and transferred charge along with the frequency at different amplitudes are demonstrated in Figure 4e,f. The relationship between the voltage or transferred charge and amplitude was also irregular.

Figure 4g shows the linear relationship between the short-circuit current and the vibration frequency at a fixed amplitude of 1 mm. Furthermore, there was also a linear relationship between the short-circuit current and the vibration amplitude from 0.5 to 9 mm under a vibration frequency of 20 Hz, which can be seen in Figure 4h. Thus, this shows the potential of the SRS-TENG to be a vibration frequency and amplitude sensor in the frequency range of 5–90 Hz and amplitude range of 0.5–9 mm.

Moreover, the output performance of the SRS-TENG under fixed acceleration was also studied, which is shown in Figure 4i. It can be seen that the short-circuit current reached 0.65 μA at 25 Hz and then decreased with the fixed acceleration of 20 m/s^2^. This is because 25 Hz is the resonant frequency at which the silicone rubber strip has the best contact with the upper and lower electrodes under this acceleration.

**Figure 5:**
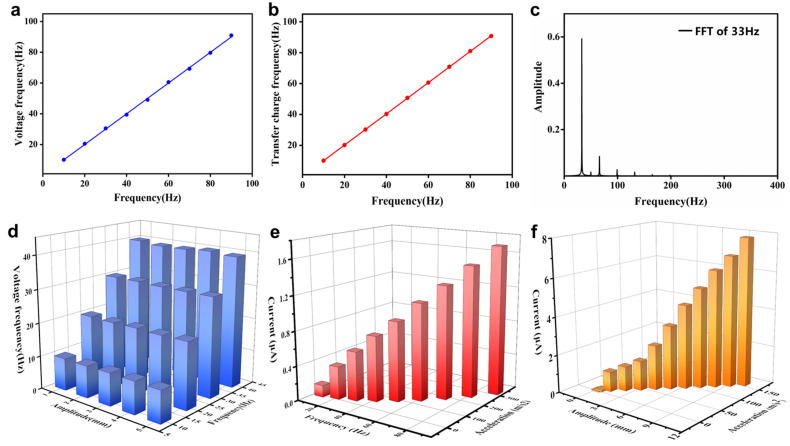
Vibration sensing performance of the SRS-TENG. (**a**) Linear relationship between FFT of voltage signal and vibration frequency with the vibration amplitude of 1 mm. (**b**) Linear relationship between FFT of transferred charge signal and vibration frequency with the vibration amplitude of 1 mm. (**c**) The FFT result of voltage signal with the vibration frequency of 20 Hz and amplitude of 1 mm. (**d**) 3D relationship between the FFT result of voltage signal and vibration frequency with different fixed amplitude. (**e**) Relationship between short-circuit current, vibration frequency and acceleration with the fixed amplitude of 1 mm. (**f**) Relationship between short-circuit current, vibration amplitude and acceleration with the fixed frequency of 20 Hz.

Through the analysis of the above test results, the voltage and transferred charge were not found to show a linear relationship with amplitude or frequency. However, after the fast Fourier transform (FFT) of the voltage and transferred charge, the FFT results showed linearity with different vibration frequencies, as demonstrated in Figure 5a,b. The FFT result of the vibration frequency of 33 Hz under the vibration amplitude of 1 mm is illustrated in Figure 5c.

The relationship between the FFT results of the voltage signal and frequency under different amplitudes is shown in Figure 5d, which provides another way to detect the vibration frequency. Figure 5e,f show the 3D relationship between the output current, vibration frequency (or amplitude) and maximum acceleration at an amplitude of 1 mm (or frequency of 20 Hz). The results show that the output current increased with increasing vibration acceleration and vibration frequency or amplitude, so the SRS-TENG has the potential to be used as a multifunctional vibration sensor. From the above observation, the SRS-TENG can be used to accurately detect vibration frequencies from 5 to 90 Hz, vibration amplitudes of 0.5–9 mm, and accelerations from 0.5 to 319.8 m/s^2^. The amplitude display system, vibration monitoring and alarm system are demonstrated with a detailed discussion in the next chapter.

### 2.4. Demonstration

**Figure 6:**
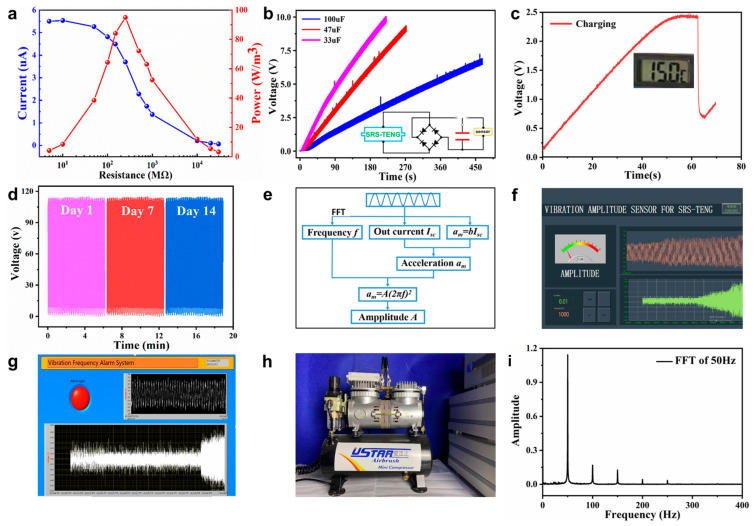
The demonstration of the SRS-TENG as a vibration energy harvester and self-powered vibration sensing and alarm system. (**a**) Current and output power density of the SRS-TENG under a vibration frequency of 30 Hz and an amplitude of 2 mm. (**b**) Voltage curves of different capacitors charged by SRS-TENG. (**c**) Voltage curve of the capacitor charged by the SRS-TENG powering a temperature sensor. (**d**) Robustness performance of the SRS-TENG after 14 days test. (**e**) The logic diagram of SRS-TENG for vibration amplitude and frequency sensing. Demonstration of (**f**) vibration amplitude sensing and (**g**) vibration frequency alarm system. (**h**) The SRS-TENG mounted on a commercial air compressor as a vibration sensor and (**i**) the FFT result of the voltage signal of the SRS-TENG.

Figure 6 demonstrates the performance of the SRS-TENG to be an energy harvester and a self-powered vibration sensing and alarm system. Based on the test results, the energy-harvesting performance demonstration is indicated in Figure 6a–c. The output performance of the SRS-TENG was determined by mounting the SRS-TENG on an electrodynamic shaker at the working frequency of 30 Hz and an amplitude of 2 mm. The maximum power density 94.95 W/m^3^ was achieved at the load resistance of 250 MΩ, as illustrated in Figure 6a. Capacitors were charged by the SRS-TENG through the rectifying circuit shown in the inset in Figure 6b. The circuit was adopted to change the alternating current to a direct current, which can be used to power the sensor and is useful for energy harvesting and self-powered sensing based on the SRS-TENG. Different capacitor charging performances are exhibited in Figure 6b; the 33, 47 and 100 µF capacitors could be charged to 5 V in 90, 130 and 315 s, respectively. Moreover, as depicted in Figure 6c and Video S2 in the Supplementary Materials, the model is capable of powering a commercial temperature sensor after charging the capacitor in 60 s, which demonstrates the practical application of the SRS-TENG as a power source. Furthermore, the SRS-TENG could also light up more than 100 LEDs, as shown in Video S3 in the Supplementary Materials, exhibiting its excellent performance in vibration energy harvesting. In addition, owing to the unique design of the SRS-TENG, the soft contact between the strip and electrode means that the SRS-TENG qualifies for the features of robustness and durability depicted in Figure 6d. The output performance was almost the same as the original state after 14 days.

From above analysis, the SRS-TENG is capable of acting as a multifunctional vibration sensor for monitoring real-time vibration acceleration, amplitude and frequency. As shown in Figure 5f, the quantitative relationship of maximum acceleration (*a_m_*), and output current (*I_sc_*) is linear under a fixed vibration frequency, which can be determined as *a_m_* = *bI_SC_*, where *b* is a constant. Then, by substituting the real-time output current *I_SC_* to *a_m_* = *bI_SC_*, the maximum acceleration *a_m_* is obtained, which can further be used to calculate the amplitude using Equation (2), where the frequency *f* can be easily obtained by the FFT result. Figure 6e indicates the logic diagram of the SRS-TENG as a self-powered vibration frequency detection and alarm system and an amplitude sensor. The practical application of the SRS-TENG to be an amplitude sensor is demonstrated in Figure 6f and Video S4 in the Supplementary Materials, in which the amplitude is displayed on the screen. Figure 6g and Video S5 in the Supplementary Materials show the application scenario of the SRS-TENG acting as the frequency alarm system, and when the frequency exceeds the limit, the red light illuminates. Here, the vibration frequency changes from 30 Hz to 40 Hz; after it reaches 40 Hz, which is set as the frequency limit, the alarm is triggered instantly. Furthermore, the SRS-TENG is mounted on a commercial air compressor to monitor its working condition, as shown in Figure 6h. The air compressor is a two-stage type, with a revolution speed of 1500 rpm. As illustrated in Figure 6i, the FFT result of the voltage signal was about 50 Hz, which is equal to the theoretical working frequency of the air compressor calculated by the equation *f_ac_* = (1500 × 2)/60 = 50 Hz.

## 3. Conclusions

In summary, this work proposes a novel silicone rubber strip-based triboelectric nanogenerator, which can work in different modes, as a broadband vibration energy harvester and multifunctional self-powered vibration sensor. The SRS-TENG is composed of two conductive aluminum electrodes supported by PLA and a silicone rubber strip with two ends fixed at half the height of the rectangular prism. The SRS-TENG is capable of acting as a vibration energy harvester and self-powered sensor in the vibration frequency range of 5–90 Hz and amplitude range of 0.5–9 mm. The SRS-TENG has obvious broadband advantages compared to spring-assisted TENGs, and a lower limit starting advantage than the bouncing-ball TENGs. In addition, the power density of the SRS-TENG can achieve 94.95 W/m^3^, and it can light up more than 100 LEDs and power a commercial temperature sensor after charging the capacitors. Moreover, because of the soft contact between the strip and electrodes, the SRS-TENG is robust and has hardly performance degradation, even after 14 days. Meanwhile, the SRS-TENG can be used as a multifunctional vibration sensor, for example using the vibration frequency, amplitude and acceleration sensors. Furthermore, the SRS-TENG can be used to monitor vibration amplitude and emit vibration frequency alarms after processing. The amplitude value and frequency alarm can be displayed on the human–machine interface based on LabView. Finally, it can be successfully applied on the working frequency monitoring of an air compressor. In conclusion, the SRS-TENG is capable of acting as a sustainable broadband power source for sensor nodes and a self-powered multifunctional vibration sensor and vibration alarm system in various fields.

## 4. Experimental Section

### 4.1. Fabrication of the SRS-TENG

Fabrication of the SRS-TENG: The schematic diagram of the new design is presented in Figure 1b. The device consists of a rectangular prism which was printed by a 3D printer. The aluminum layers with a thickness of 0.2 mm were attached on the top and bottom PLA housing as electrodes in addition to the positive triboelectric material. In the middle of the rectangular prism, the silicone rubber strip was fixed on both ends of the rectangular prism at half-height.

The silicone rubber strip was prepared using Eco-flex 30 silicone rubber part (A & B) (Smooth-On, Macungie, PA, USA) mixing of an equal ratio. A spin-coater was applied to spin the well-mixed silicone rubber on the surface of fine sandpaper to carry out the surface modification of the silicone rubber membrane. The desired thickness of the silicone rubber strip was obtained by adjusting the rotation speed, acceleration, and rotating time of the spin-coater, which is shown in Table S1. Finally, the silicone rubber was dried in a constant temperature vacuum furnace for 12 h at the temperature of 40 °C.

### 4.2. Observation of Movement of the Silicone Rubber Strip

The movement of the silicone rubber strip was observed by applying a high-speed camera (FATCAM Mini UX50, Photron, Tokyo, Japan) and simulations were performed by COMSOL Multiphysics software (COMSOL Inc., Stockholm, Sweden).

### 4.3. Measurement of the Electric Output

The SRS-TENG was attached to an electrodynamic shaker (JZK-20, SINOCERA, Suzhou, China) driven by an amplifier (YE5852, SINOCERA, Suzhou, China) after receiving the vibration signal from an adjustable functional signal generator (YE1311, SINOCERA, Suzhou, China). The acceleration was detected by a commercial single-axis accelerometer (KS96.100, MMF, Radebeul, Germany), analyzed in a Dynamic Signal Analyzer (Inelta, Chengdu, China), and then displayed in DASP software (Inelta, Chengdu, China). The electric output signals, including open-circuit voltage, short-circuit current, and transferred charge, were measured by an electrometer (Keithley 6514, Tektronix, Beaverton, OR, USA) and sent to the LabView-based computer through a DAQ device. The FFT process was carried out by the Origin software.

## Data Availability

The data presented in this study are available on request from the corresponding author.

## Supplementary Materials

The following supporting information can be downloaded at: https://www.mdpi.com/article/10.3390/nano12081248/s1, Figure S1: The working mechanism of the N-C model. Figure S2: Testing system for SRS-TENG. Figure S3: Performance comparison between the SRS-TENGs with double electrodes and a single electrode. Figure S4: Performance comparison between the SRS-TENGs with or without surface treatment of the strip. Figure S5: Performance comparison among the SRS-TENGs with different strip widths of 22, 30 and 42 mm. Figure S6: Performance comparison among the SRS-TENGs with different strip thicknesses of 0.48, 0.86, and 1.2 mm. Figure S7: Performance comparison among the SRS-TENGs with different air gaps of 5, 10 and 15 mm. Table S1: Spin-coater parameter setting for different strip thickness. Video S1: Strip moving status under vibration frequencies of 10, 30 and 50 Hz with an amplitude of 1 mm. Video S2: Powering of a temperature sensor by the SRS-TENG. Video S3: Lighting up 112 LEDs by the SRS-TENG. Video S4: Demonstration of amplitude sensing. Video S5: Demonstration of vibration alarm system.
