# Supplementary material for: A Robust Silicone Rubber Strip-Based Triboelectric Nanogenerator for Vibration Energy Harvesting and Multi-Functional Self-Powered Sensing
Source: Nanomaterials (Basel). 2022 Apr 7;12(8):1248. doi: 10.3390/nano12081248 (PMC9030836; doi:10.3390/nano12081248)
Supplement: Supplementary file 1 [file nanomaterials-12-01248-s001.zip › nanomaterials-1630271-Supplementary Materials.pdf]

nanomaterials

Supplementary Materials

A Robust Silicone Rubber Strip-Based Triboelectric Nanogenerator for Vibration Energy Harvesting and Multi-Functional Self-Powered Sensing

Taili Du [1,2,†], Bin Ge [1,†], Anaeli Elibariki Mtui [1,†], Cong Zhao [1], Fangyang Dong [1,2], Yongjiu Zou [1,2], Hao Wang [1], Peiting Sun [1,2,*] and Minyi Xu [1,*]

1 Dalian Key Lab of Marine Micro/Nano Energy and Self-Powered Systems, Marine Engineering College, Dalian Maritime University, Dalian 116026, China; dutaili@dlmu.edu.cn (T.D.); gebin@dlmu.edu.cn (B.G.); mtyellie93@gmail.com (A.E.M.); zhaocong@dlmu.edu.cn (C.Z.); dongfangyang@dlmu.edu.cn (F.D.); zouyj0421@dlmu.edu.cn (Y.Z.); hao8901@dlmu.edu.cn (H.W.)
2 Collaborative Innovation Research Institute of Autonomous Ship, Dalian Maritime University, Dalian 116026, China
* Correspondence: sunptg@dlmu.edu.cn (P.S.); xuminyi@dlmu.edu.cn (M.X.)
† These authors contribute equally to this work.

Citation: Du, T.; Ge, B.; Mtui, A.E.; Zhao, C.; Dong, F.; Zou, Y.; Wang, H.; Sun, P.; Xu, M. A Robust Silicone Rubber Strip-Based Triboelectric Nanogenerator for Vibration Energy Harvesting and Multi-Functional Self-Powered Sensing. *Nanomaterials* **2022**, *12*, 1248. https://doi.org/10.3390/10.3390/nano12081248

Academic Editor: Alessia Irrera

Received: 23 February 2022
Accepted: 4 April 2022
Published: 7 April 2022

Publisher's Note: MDPI stays neutral with regard to jurisdictional claims in published maps and institutional affiliations.

Copyright: © 2022 by the authors. Licensee MDPI, Basel, Switzerland. This article is an open access article distributed under the terms and conditions of the Creative Commons Attribution (CC BY) license (https://creativecommons.org/licenses/by/4.0/).

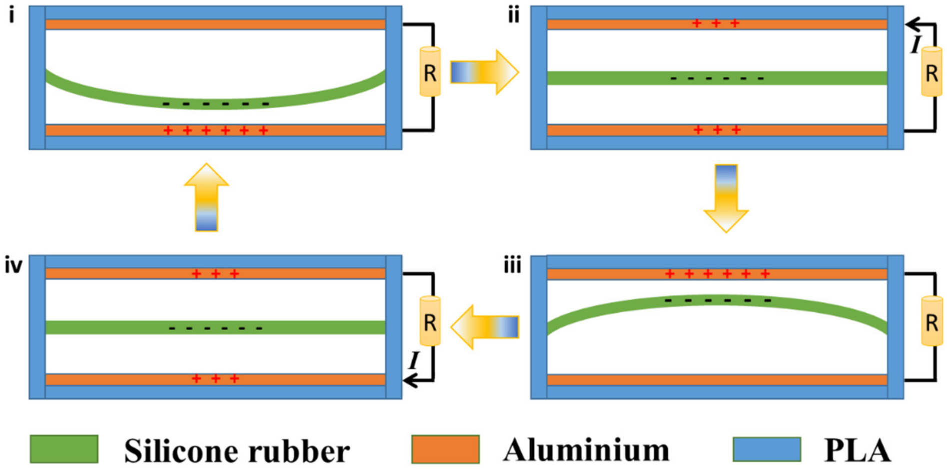

Figure S1. The working mechanism of the N-C model.

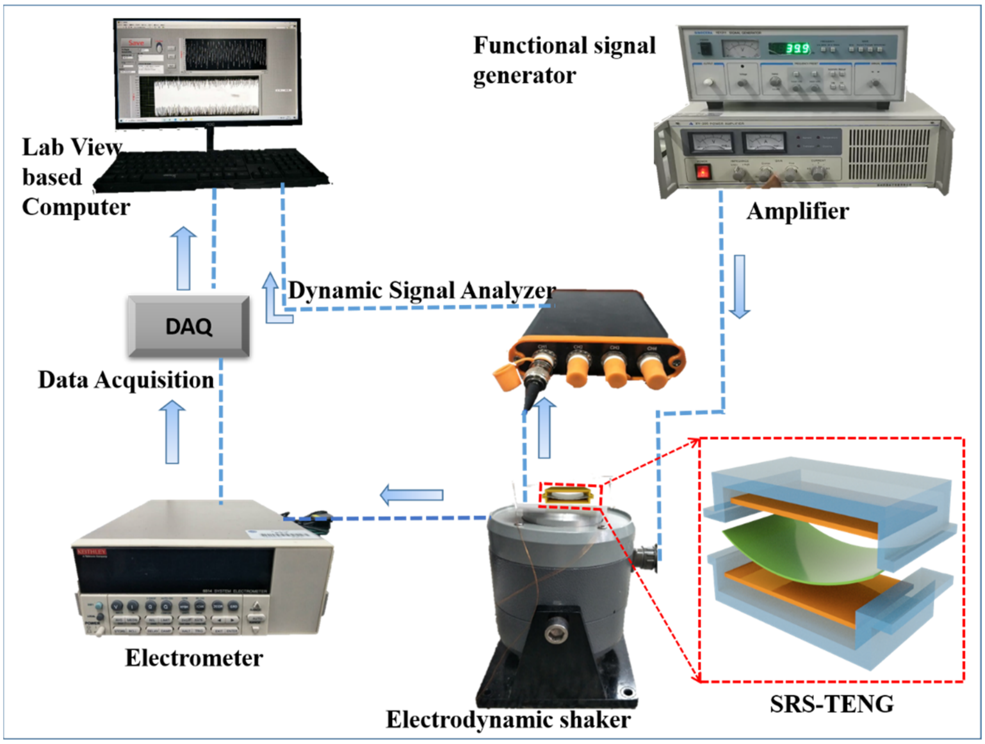

Figure S2. Testing system for SRS-TENG.

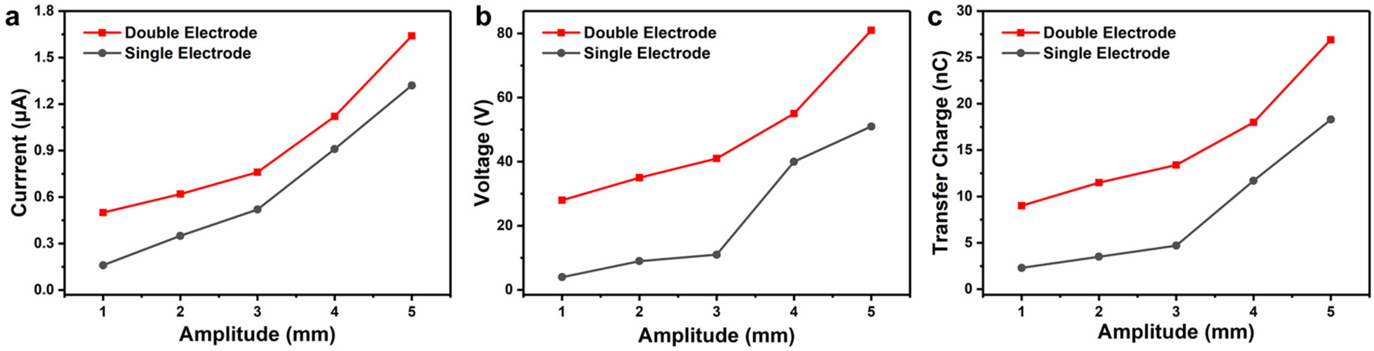

Figure S3. (**a**) Current, (**b**) voltage; (**c**) transfer charge performance comparison between the SRS-TENGs working at double electrode and single electrode mode.

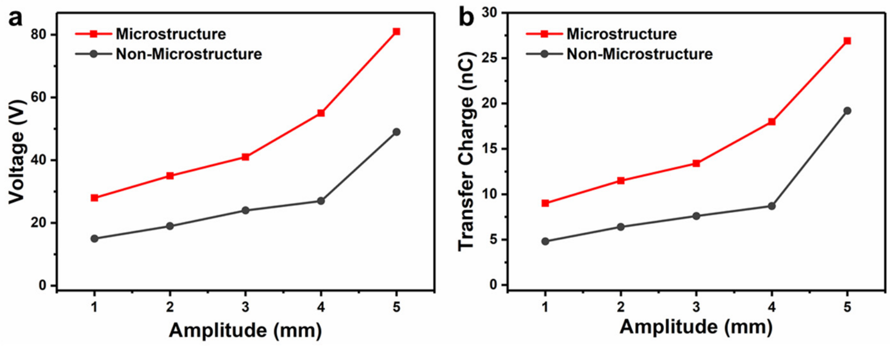

Figure S4. (**a**) Voltage and (**b**) transfer charge performance comparison between the SRS-TENGs with or without surface treatment of the strip.

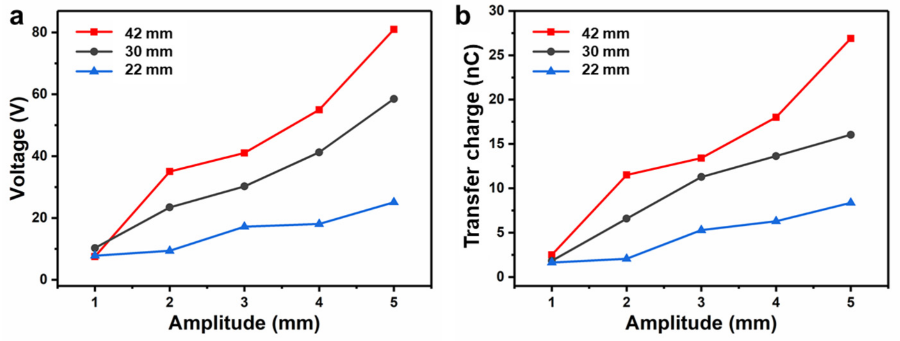

Figure S5. (**a**) Voltage and (**b**) transfer charge performance comparison among the SRS-TENGs with different widths of 22, 30 and 42 mm.

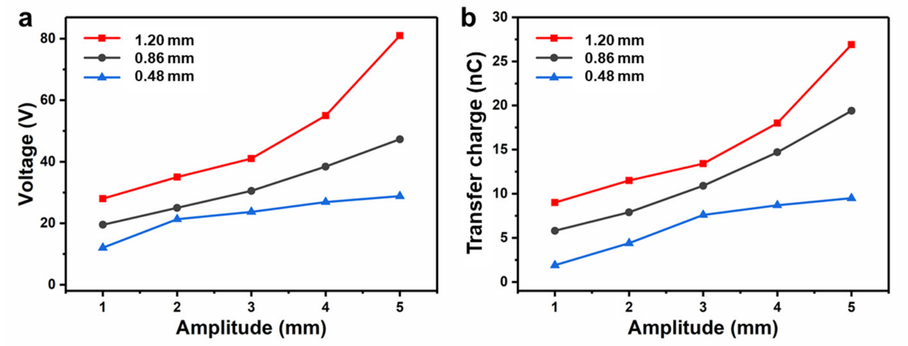

Figure S6. (**a**) Voltage and (**b**) transfer charge performance comparison among the SRS-TENGs with different thicknesses of 0.48, 0.86 and 1.2 mm.

Table S1. Spin-coater parameter setting for acquiring strip thickness.

	Acceleration (m/s^2)	Speed (m/s)	Time (s)	Thickness (mm)
1	1000	200	15	0.48
2	1000	200	10	0.86
3	1000	200	5	1.20

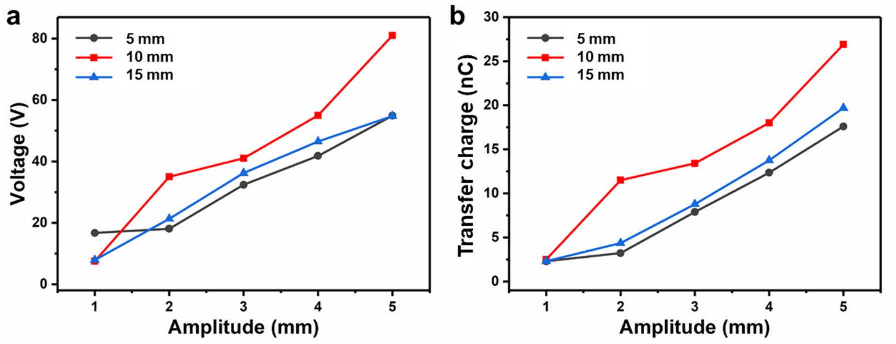

Figure S7. (**a**) Voltage and (**b**) transfer charge performance comparison among the SRS-TENGs with different air gaps of 5, 10 and 15 m.

Video S1. Strip moving status under vibration frequencies of 10, 30 and 50 Hz with constant amplitude of 1 mm.

Video S2. Powering a temperature sensor by the SRS-TENG.

Video S3. Lighting up 112 LEDs by the SRS-TENG.

Video S4. Demonstration of amplitude sensing.

Video S5. Demonstration of vibration alarm system.
